# Simulation and experimental study on the stability and comfortability of the wheelchair human system under uneven pavement

**DOI:** 10.3389/fbioe.2023.1279675

**Published:** 2023-11-02

**Authors:** Haitao Luo, Xuan Cao, Yuming Dong, Yuxin Li

**Affiliations:** ^1^ State Key Laboratory of Robotics, Shenyang Institute of Automation, Chinese Academy of Sciences, Shenyang, China; ^2^ Institutes for Robotics and Intelligent Manufacturing, Chinese Academy of Sciences, Shenyang, China; ^3^ School of Mechanical Engineering, Shenyang Ligong University, Shenyang, China; ^4^ School of Mechanical Engineering, Shenyang University of Technology, Shenyang, China; ^5^ University of Chinese Academy of Sciences, Beijing, China

**Keywords:** comfort evaluation, wheelchair–body system, the rate of psychological distress, roll angle and pitch angle, experimental study

## Abstract

With the improvement in the level of science and technology and the improvement of people’s living standards, the functions of traditional manual wheelchairs have been unable to meet people’s living needs. Therefore, traditional wheelchairs have been gradually replaced by smart wheelchairs. Compared with traditional wheelchairs, smart wheelchairs have the characteristics of light operation and faster speed. However, when driving on some complex road surfaces, the vibration generated by the bumps of the motorcycle will cause damage to the human body, so wheelchairs with good electric power and stability can better meet the needs of people and make up for their travel needs. Based on the traditional vehicle stability analysis method, the mathematical theory of roll stability and pitch stability of the wheelchair–human system was established. We built a multi-body dynamics model with human skeleton and joint stiffness based on the multi-body dynamics method. The functioning of the wheelchair–human system was simulated and analyzed on the ditch, step, and combined road. The acceleration and Euler angle changes of the human head, chest, and wheelchair truss position were obtained, and the data results were analyzed to evaluate the stability and comfort of the system. Finally, a wheelchair test platform was built, and the road driving test was carried out according to the simulation conditions to obtain the system acceleration and angle data during the driving process. The simulation analysis was compared to verify the accuracy of the multi-body dynamics method, and the stability and comfort of the system were evaluated.

## 1 Introduction

In today’s society, the problem of population aging is increasing, and the number of people with disabilities is also increasing. With the increase in the number of disabled people in the world, the number of disabled people with movement disorders and severe paralysis accounts for the vast majority. Therefore, as a travel tool for the elderly and disabled people with limited mobility, electric smart wheelchairs have great medical value in today’s society.

Smart wheelchairs generally come with features for easy control and outdoor navigation. For example, a wheelchair operated in real-time can switch modes between a joystick control mode and a head gesture control mode according to the user’s requirements, and a smart wheelchair can be commanded through gestures (Pathan et al., 2020; Sharma and Mathur, 2021). It is also possible to enable the smart wheelchair to realize the function of outdoor navigation through sensing information and preloaded maps of the building and its surrounding outdoor areas and based on the ultrasonic obstacle avoidance system to increase the obstacle avoidance function of the smart wheelchair, that is, to install ultrasonic sensors on the smart wheelchair. It is used to detect obstacles in the path of the wheelchair (Luo et al., 2021; Yi et al., 2023), and it is the command recognition to complete obstacle avoidance and obstacle surmounting (Elkodama et al., 2020).

However, when a smart wheelchair travels on some complex surfaces, the vibrations caused by the bumps on the road will cause harm to the human body (Pajic et al., 2017) and even cause the wheelchair to become unstable due to improper operation, resulting in more serious consequences. Therefore, it is necessary to ensure that the wheelchair can still ensure the comfort and stability of the wheelchair when driving on complex roads and obstacle roads. This is the basis for safeguarding the mobility needs of the elderly and people with disabilities.

Damien Pavec et al. established a human body-wheelchair stability dynamics model, conducted extended research on the human body stability of the wheelchair during acceleration and braking, and proposed that the lower profile of the wheelchair seat has a greater impact on the lateral stability of the human body, providing guidance for optimizing wheelchair design (Maeda and Mansfield, 2005). A new method for numerical simulation of wheel–surface interaction (Lin, 2020), which combines the finite element method with the discrete element method, rigid body dynamics, and advanced wheel–surface friction model, can better evaluate the longitudinal and lateral forces of wheels at various angles of attack (Czapla and Pawlak, 2022). By establishing a theoretical dynamic vehicle model with seven degrees of freedom, the control stability of the vehicle is simulated, the influence of road surface changes on the control stability of the vehicle is analyzed, and the vehicle handling stability is also analyzed (Bi-bao and Tao, 2021; Li et al., 2021). Based on the vehicle stability theory, the vehicle’s dynamic stability is analyzed, and the wheelchair rollover is pre-warned and controlled by the MFC (Paddan and Griffin, 2002; Burkhard et al., 2021). Items of seat comfort use were analyzed using item response theory (Menegon et al., 2019). When the wheelchair passes through complex road surfaces such as steps and ditches, and generates strong bumps, the vibrations generated will cause harm to the human body. Therefore, wheelchairs with good passability and stability can better provide riding comfort and meet people’s travel needs. The ability to improve occupant vibration comfort has been discussed in Kim et al. (2020). Preliminary studies show that the current ISO-2631 standard achieves good results in objectifying and building an extended model in order to be able to robustly objectify ride comfort; experiments show that the extended model can create comparable or more accurate comfort than the ISO-2631 degree value (Burkhard et al., 2021). The comfort of the human seat system is explored, and the pressure distribution of the driver's human body and the influence of perceived discomfort are explored (Dong et al., 2019; Tahir et al., 2020; Lantoine et al., 2022). The impact of the angle of the seat back on the driver's comfort is also significant (Mačužić and Lukić, 2020), and it is necessary to evaluate whether the seat posture is appropriate through comfort and user experience (Caballero-Bruno et al., 2022). Furthermore, by changing the angle of the prone position, the effects of different postures on the average pressure, maximum pressure, and pressure area of the hip were explored (Kim et al., 2023). The vibration transmission of the wheelchair has a particularly obvious impact on the head and spine of the human body. A three-dimensional numerical model of the human spine specially used for vibration research is used. The model was constructed using multibody dynamics techniques (Valentini and Pennestrì, 2016; Song et al., 2019). By monitoring the vibrations transmitted to the body trunk, the proposed method can estimate posture changes and periodic fluctuations during wheelchair propulsion with high reproducibility (Takeo et al., 2023). It can be seen that the stability of the vehicle during driving is very important to the comfort of the smart wheelchair occupant. Vehicles are evaluated through obstacle avoidance tests to determine whether they can maintain human comfort when traversing obstacles (Parra et al., 2022).

The occupant is mainly subjected to vertical vibration excitation during the driving process of the vehicle. The study of the biomechanical characteristics of the vertical vibration of the sitting human body has always been a hot research topic, mainly including experimental research and theoretical modeling of the vertical vibration characteristics of the sitting human body. A KC mass system model (a classical stiffness damping model) was established with parallel double degrees of freedom and used to conduct in-depth research on the vertical characteristics of the human body at 0–10 Hz, and we also established a vertical vibration simulation device for the human sitting posture based on this research model (Suggs C W et al., 1969). Research confirms the strong correlation between vibration excitation in the vertical direction of the human body and human comfort (Zhang et al., 2018). Based on the vehicle theory, when the vehicle is driving on a complex road surface, the driver will be subjected to low-frequency (0.5–25 Hz) severe vibration, and the sustained vertical vibration will cause damage to the head and waist of the occupant (Adam et al., 2019).

At the same time, the ride comfort of the vehicle may be affected by the characteristics of the seat. The vertical vibration of the seat is related to the thickness and material of the seat. They affect the front-to-back coaxial and vertical cross-axis transmission capabilities of the seat panel and backrest. The resonant frequency of the fore–aft in-line transmissibility of the seat pan and backrest decreases with increasing foam thickness at the seat pan and backrest (Zhang et al., 2021). Sitting posture and vibration amplitude also have a significant impact on the vibration transmission rate of the seat suspension system. Experiments have found that by giving subjects vertical vibrations of different frequencies (Shu et al., 2021), they will perceive different degrees of comfort under different sitting postures and vibration amplitudes (Adam et al., 2020).

This article first establishes the stability theory of the wheelchair–human body system through the classic dynamic model theory of vehicles, conducts a theoretical analysis of the rollover stability and pitch stability of the wheelchair–human body system, and establishes an objective comfort evaluation system based on subjective psychological distress rate index. A comprehensive evaluation system for human comfort will further establish the biomechanical model of the rigid wheelchair–human body system; carry out driving simulation analysis on the system on ditches and step roads; extract the acceleration and angle changes in the human head, chest, and frame truss centroid positions and analyze the data results. The wheelchair test system platform is built to carry out the driving test under the same simulated working conditions and compare it with the multi-body dynamics simulation results to verify the accuracy of the simulation analysis. Finally, based on the stability theory and comfort evaluation system, the stability and comfort of the wheelchair–human body system under complex road conditions are evaluated.

## 2 Wheelchair—theory of human system stability

When the wheelchair is driving on a complex road surface, the bumps on the road surface will cause the wheelchair to produce left and right roll and pitch motions. When the road surface has large undulations, it may cause the risk of wheelchair rollover and dumping, and the vibration excitation is transmitted to the human body through the wheelchair. When it is serious, it will affect human comfort and cause harm to the body. Based on the theory of roll stability and pitch stability of classic vehicles, the rollover and status of the wheelchair–human body system are theoretically analyzed, and the rollover and pitch stability limits of the wheelchair–human system are obtained to provide theoretical guidance for subsequent driving tests.

In addition, the comfort level when riding in a wheelchair is evaluated based on the objective acceleration evaluation index and subjective annoyance rating index, providing a theoretical basis for subsequent simulation analysis and driving tests.

### 2.1 Wheelchair–human system rollover stability theory

**FIGURE 1:**
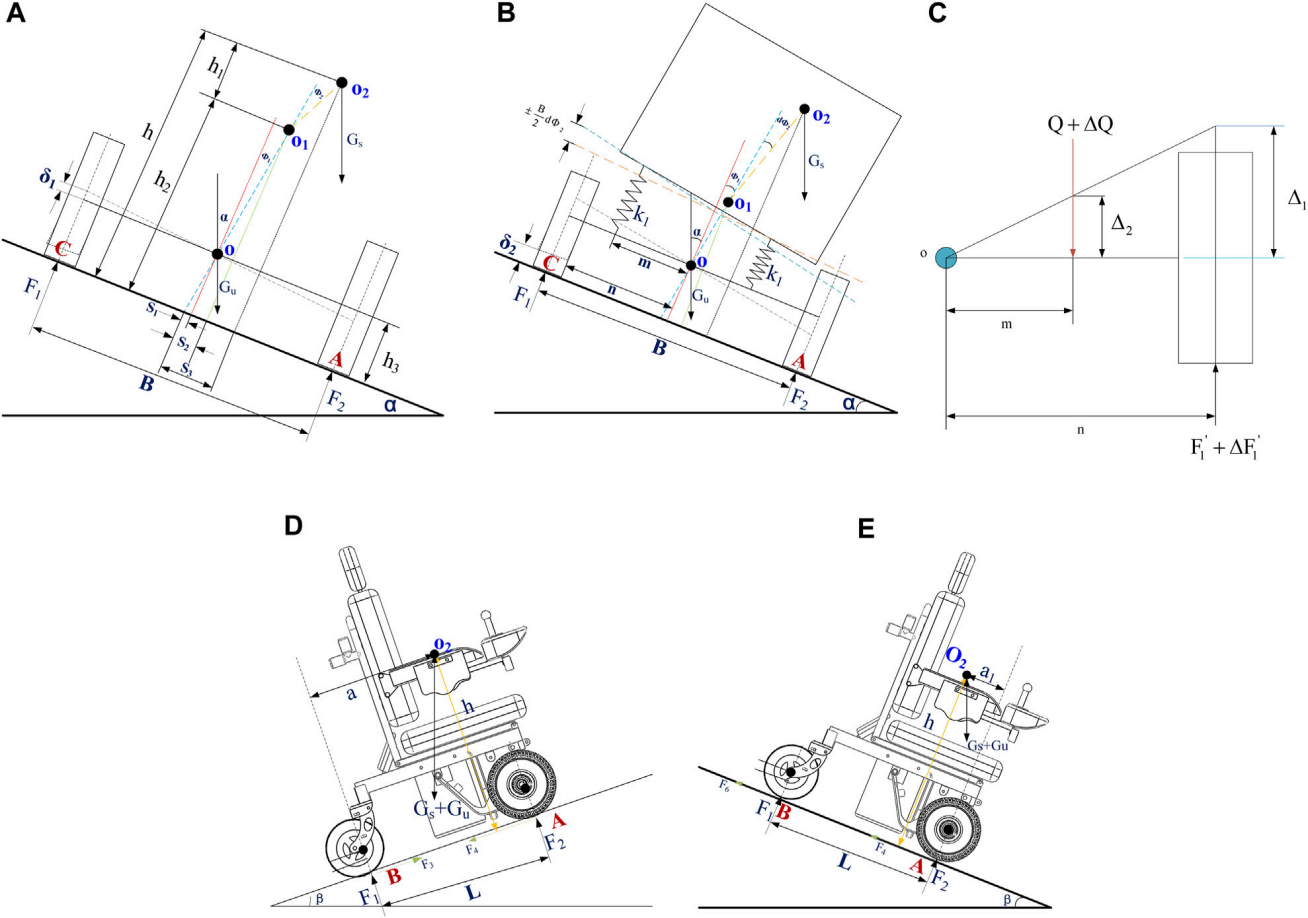
Simplified diagram of wheelchair system roll and uphill and downhill forces **(A)** Schematic representation of the wheelchair system on its side. **(B)** Analysis of equivalent spring for suspension of the wheelchair system. **(C)** Stiffness of the unilateral suspension wire of the wheelchair. **(D)** Schematic diagram of the forces on the wheelchair–human system when going uphill. **(E)** Schematic diagram of downhill force on the wheelchair–human system.

In order to maximize the accuracy of the wheelchair roll dynamics model, the influence of the two main factors, the combined angular stiffness of the independent suspension and the vertical stiffness of the tire, is considered comprehensively during the modeling process. The rollover of the wheelchair system is shown in Figure 1A.

In Figure 1A, $\delta_{1}$ is the vertical displacement of the tire caused by the unsprung mass of the wheelchair; $G_{s}$ is the sprung mass for wheelchairs; $G_{u}$ is the unsprung mass for wheelchairs; $O_{2}$ is the center of the mass point of the sprung mass of the wheelchair; $O_{1}$ is the center of roll of the wheelchair body; O is the center of the mass point of the unsprung mass of the wheelchair; B is the wheelbase of the wheelchair; $F_{1}$ is the wheelbase of the wheelchair; $F_{2}$ is the truss value of the right wheel of the wheelchair; $S_{1}$ , $S_{2}$ , and $S_{3}$ are the distances between the center of the mass of the unsprung mass, the center of roll and the center of the mass of the sprung mass, and the longitudinal plane of the symmetry of the wheelchair; is the distance from the center of mass of the sprung mass of the wheelchair to the slope; $h_{1}$ is the distance from the center of the mass of the sprung mass of the wheelchair to the roll center of the wheelchair; $h_{2}$ is the distance from the roll center of the wheelchair body to the slope; $h_{3}$ is the equivalent height of the center of mass of the unsprung mass; α is the static roll angle; $\Phi_{1}$ and $\Phi_{2}$ are the roll angular displacements of the unsprung mass and the sprung mass, respectively. The overturning moment of the sprung mass of the wheelchair is equal to the combination of the suspension of the wheelchair body called stiffness. Taking the moment about the roll center point $O_{1}$ :

$$G_{s}\sin\alpha h+G_{s}\cos\alpha \left(S_{3}-S_{2}\right)=K_{r}\Phi_{1},$$

where $K_{r}$ is the combined angular stiffness of the wheelchair suspension.

Substituting $S_{3}$ and $S_{2}$ , the relationship between $\Phi_{1}$ and $\Phi_{2}$ can be obtained as

$$\Phi_{2}=\left(\frac{K_{r}}{G_{s}\cos\alpha h}-1\right)\Phi_{1}-\tan\alpha .$$

The angular stiffness of the suspension is the elastic restoring moment received by the unit roll angle generated by the body roll. Figure 1B shows the suspension spring analysis diagram of the wheelchair when the whole vehicle is tilted.

As shown in Figure 1C, m is the distance between the suspension spring and the longitudinal symmetrical plane of the wheelchair; n is the distance between the tire and the longitudinal plane of symmetry of the wheelchair; $k_{1}$ is the suspension spring stiffness (Nm/rad); $F_{1}$ is the force of the tire on the loose side; and $F_{2}$ is the force of the tire on the tightening side. In order to obtain the combined angular stiffness of the wheelchair suspension, we first need to calculate the linear stiffness of the one-sided suspension of the wheelchair.

As shown in Figure 1C, $F_{1}$ and $\Delta F_{1}$ are the forces and increments on one side of the tire, respectively; Q and ΔQ are the spring force and increment of the suspension spring, respectively; $\Delta_{1}$ is the vertical displacement increment produced by the tire; and $\Delta_{2}$ is the vertical displacement increment produced by the suspension spring. We know that the stiffness coefficient of the suspension spring is $k_{s}$ . From this, according to the aforementioned figure, the bus stiffness of the wheelchair suspension can be obtained as

$$k_{l}=2k_{s}{\left(m/n\right)}^{2}.$$

When the wheelchair body has a roll angle increment of $d\Phi_{2}$ , the deformation produced by the equivalent spring is $\pm \frac{B}{2}d\Phi_{2}$ . The combined angular stiffness $k_{r}$ of the suspension of the wheelchair is obtained as

$$k_{r}=k_{s}{\left(\frac{Bm}{n}\right)}^{2}.$$

Assuming that the stiffness of the wheelchair tire is linear, the deformation of the tires on both sides of the car on the horizontal road is set to $\Delta_{0}$ ; then:

$$\Delta_{0}=\frac{\left(G_{u}+G_{s}\right)}{2k_{t}},$$

where $k_{t}$ is the vertical linear stiffness of the tire.

When the wheelchair is on a slope with a gradient of α , the load on the left and right wheels will be transferred under the action of gravity, causing the tire to deform. Assuming the deformation of the tire is Δ , we obtain

$$F_{2}=k_{t}\Delta =k_{t}\left(\Delta_{0}+\delta_{1}\right).$$

By decomposing the force system along the normal direction of the slope, the synthesis of the tire support force is equal to the synthesis of gravity components:

$$F_{1}+F_{2}=\left(G_{u}+G_{s}\right)\cos\alpha .$$

According to the geometric relationship in Figure 1A, it can be seen that

$$\delta_{1}=B\Phi_{1}/2.$$

By combining the aforementioned formulas, the normal force of the tires on both sides in contact with the slope can be obtained as

$$\left\{\begin{array}{l} F_{1}=\left(G_{u}+G_{s}\right)\cos\alpha /2-k_{t}B\Phi_{1}/2\\ F_{2}=\left(G_{u}+G_{s}\right)\cos\alpha /2+k_{t}B\Phi_{1}/2 \end{array}\right.,$$

where $F_{1}$ is the stress on the tire on the loose side and $F_{2}$ is the force on the tire on the compaction side.

Figure 1A shows the moment of contact point A between the tire and the slope, and we get

$$F_{1}B+G_{s}\sin\alpha h+G_{u}\sin\alpha h_{3}=G_{s}\cos\alpha \left(\frac{B}{2}-S_{3}\right)+G_{u}\cos\alpha \left(\frac{B}{2}-S_{1}\right).$$

Simultaneously simplifying the aforementioned formulas, it can be seen that the vertical linear stiffness of the tire is

$$K_{t}=\frac{2\left(G_{u}h_{3}+G_{s}h\right)\left(\sin\alpha +\cos\alpha \Phi_{1}\right)+2G_{s}h_{1}\cos\alpha \Phi_{2}}{B^{2}\Phi_{1}}.$$

When the slope reaches the maximum roll angle $\alpha_{1}$ of the wheelchair, the tires on the upper side of the slope just leave the slope. $F_{1}=0$ , which can be obtained from the following resultant force formula:

$$\frac{\left(G_{u}+G_{s}\right)}{2}\cos\alpha =\frac{K_{t}B\Phi_{1}}{2},$$

Combining the aforementioned relational expressions to eliminate $\Phi_{u}$ , we obtain

$$\left(G\cos^{2}\alpha_{1}+Bk_{t}\sin\alpha_{1}\right)\left(h_{g}G+\frac{G_{s}h_{1}}{\frac{k_{r}}{G_{s}h\cos\alpha_{1}}-1}\right)=\frac{1}{2}k_{t}B^{2}G\cos\alpha_{1},$$

where $G=G_{u}+G_{s}$ is the total weight of the vehicle and $h_{g}=\left(G_{u}h_{3}+G_{s}h\right)/G$ is the equivalent center-of-mass height of the whole vehicle.

The aforementioned formula is the relationship between the maximum roll stability angle $\alpha_{1}$ of the vehicle, the combined angular stiffness $K_{r}$ of the independent suspension, and the vertical stiffness $K_{t}$ of the tire, which can be obtained by substituting the relevant geometric and structural parameters of the system.

### 2.2 Wheelchair–human system pitch stability theory

In the process of driving a wheelchair, the user usually passes through different rough and bumpy roads. When driving at a constant speed or accelerating on a sloped road, the wheelchair will roll over and cause accidents. Because the wheelchair usually travels at a constant speed and the speed is relatively slow, the pitch stability of the wheelchair–human body system is considered in this paper when traveling at a constant speed. In the pitch stability calculation, the suspension system and tire stiffness of the wheelchair have little influence on the stability of the pitch process during the pitch motion, so this paper ignores the influence of the tire stiffness and the wheelchair suspension system when calculating the pitch stability angle.

The force diagram of the system when it goes uphill at a constant speed is shown in Figure 1D. $G_{s}+G_{u}$ is the gravitational force on the system, $F_{1}$ is the vertical reaction force of the slope road on the rear wheel of the wheelchair, $F_{2}$ is the vertical reaction force of the road on the rear wheel of the wheelchair, $F_{3}$ is the traction force generated by the wheelchair when it is running at a constant speed, $F_{4}$ is the rolling resistance of the wheelchair tire, a is the distance from the center of mass of the wheelchair–human body system to the axis of the rear wheel of the wheelchair, h is the height of the center of the mass of the system, L is the wheelbase of the front and rear axles of the wheelchair, and β is the slope angle.

The moment balance equation is established through the contact point between the rear wheel of the wheelchair and the road surface. In order to avoid the system tipping over during the uphill process, we obtain

$$F_{2}=\frac{a\left(G_{s}+G_{u}\right)\cos\beta -h\left(G_{s}+G_{u}\right)\sin\beta }{L}\geq 0$$

$$\beta \leq \arctan\frac{a}{h}.$$

So the maximum pitch angle of the wheelchair when going uphill is

$$\beta_{\lim}=\arctan\frac{a}{h}.$$

It can be seen from the aforementioned formula that the maximum pitch angle of the system when going uphill at a constant speed is related to the distribution of the center of the mass of the wheelchair–human body system. There will also be changes. In order to prevent the wheelchair from sliding down when going uphill, it is necessary to consider the maximum slip angle when driving on a slope, where $\mu_{1}$ is the rolling friction coefficient between the wheelchair tire and the road surface, and the conditions to ensure that the system does not slip downward are as follows.

$$F_{3}=\mu_{1}F_{1}\geq F_{4}+\left(G_{s}+G_{u}\right)\sin\beta =\mu_{1}F_{2}+\left(G_{s}+G_{u}\right)\sin\beta .$$

$$F_{1}^{\prime }+F_{2}^{\prime }=\left(G_{s}+G_{u}\right)\cos\beta .$$

Combining the two aforementioned formulas, the maximum slip angle of the system when going uphill at a constant speed is

$$\beta_{1}=\arctan\frac{2\mu_{1}a-\mu_{1}L}{2h\mu_{1}-L}.$$

Based on the aforementioned formula, the slip angle of the system when going uphill at a constant speed is related to the distribution of the center of mass of the system, the wheelbase of the front and rear wheels of the wheelchair, and the rolling friction coefficient between the tire and the road surface. The maximum pitch angle of the system when going downhill is the same as when going uphill, but the slip angle of the system is also affected by the rolling resistance of the wheelchair tires during this process.

Figure 1E shows the force diagram of the system when the wheelchair goes downhill, the rolling resistance of the rear tire of the wheelchair in the figure is $F_{6}$ , the coefficient of static friction between the tire and the road is $\mu_{2}$ , and the conditions to ensure that the wheelchair–body system does not slip during this process are

$$F_{4}+F_{6}=\mu_{1}F_{2}+\mu_{2}F_{1}\geq \left(G_{s}+G_{u}\right)\sin\beta .$$

Based on the contact point $O_{1}$ of the front wheel with the road surface and from the moment balance condition, we obtain

$$F_{1}L+h\left(G_{s}+G_{u}\right)\sin\beta -a\left(G_{s}+G_{u}\right)\cos\beta =0.$$

Combining the aforementioned two formulas, the slip angle of the wheelchair–human body system when going downhill at a constant speed can be obtained as

$$\beta_{2}=\arctan\frac{\left(\mu_{1}-\mu_{2}\right)a}{\left(\mu_{1}-\mu_{2}\right)h-L}.$$

It can be seen from the aforementioned formula that the slip angle of the wheelchair when going downhill at a constant speed is related to the position distribution of the center of the mass of the system, and the static friction coefficient and rolling friction coefficient between the tire and the road surface are related to the wheelbase of the front and rear wheels of the wheelchair.

## 3 Wheelchair–human system comfort evaluation system

### 3.1 ISO-2631-1 evaluation index

**FIGURE 2:**
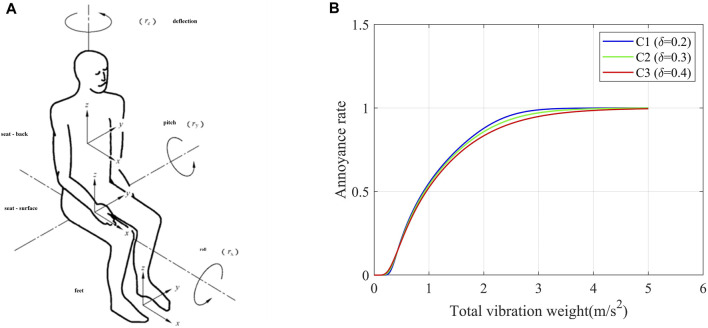
Human vibration model annoyance rate curves. **(A)** Human vibration model. **(B)** Human annoyance rate curves in the driving environment.

When evaluating the comfort of the human body in a wheelchair, the vibration model of the human sitting posture consists of the linear vibration and angular vibration in three directions of the chest, head, and feet at the contact points of the wheelchair seat and pedals. As shown in Figure 2A, the model has a total of three contact points and 12 axial vibrations. Since the wheelchair does not involve steering conditions and the driving speed is also slow, the angular vibration of its three contact points and the axial vibration of the foot contact point are ignored, and only the linear vibration of the human chest and head is considered.

Considering that vibrations in different directions and locations have different effects on the human body, the weighted root-mean-square acceleration should be calculated by the following formula or its equivalent in the frequency domain:

$$a_{\omega }={\left[\frac{1}{T}\int_{0}^{T}a_{\omega }^{2}\left(t\right)dt\right]}^{1/2},$$

where $a_{\omega }\left(t\right)$ is obtained from the weighted acceleration $a\left(t\right)$ of the time function through the frequency weighting function $\omega \left(f\right)$ , and T is the vibration analysis time.

The total vibration of the weighted root-mean-square acceleration determined by the axial vibration of the chest and head is calculated as follows:

$$a_{v}={\left(k_{x}^{2}a_{\omega x}^{2}+k_{y}^{2}a_{\omega y}^{2}+k_{z}^{2}a_{\omega z}^{2}\right)}^{1/2},$$

where $a_{\omega x}$ , $a_{\omega y}$ , and $a_{\omega z}$ are the weighted root-mean-square value relative to the orthogonal coordinate axes x, y, and z; and $k_{x}$ , $k_{y}$ , and $k_{z}$ are the direction factors.

The total vibration value $a_{v}$ is used for comfort evaluation. For a seated human body, the horizontal direction factor is 1.4, and the vertical direction factor is 1.0. Table 1 shows the relationship between the total vibration value $a_{v}$ and human comfort and ride comfort:

**TABLE 1:** Human comfort evaluation index.

Total vibration $a_{v}$ /( $m•s^{2}$ )	Human comfort evaluation	Wheelchair ride comfort C
<0.315	Stay comfortable	1.0
0.315∼0.630	Lightly uncomfortable	0.8
0.5∼1.0	Some uncomfortable	0.6
0.8∼1.6	Uncomfortable	0.4
1.25∼2.50	Very uncomfortable	0.2
>2.0	Especially uncomfortable	0

### 3.2 Evaluation of the human subjective mental annoyance rate

According to different environmental factors, people will adjust their psychological expectations, and people’s expectations for vibration will be reduced correspondingly in harsh vibration environments. Analyzing from the perspective of psychophysics, people’s subjective responses to vibration are uncertain, which is mainly due to the ambiguity caused by the unclear concept of the judgment standard of human subjective response and the sensitivity of people to vibration stimulation. Randomness is caused due to degree differences.

Fechner’s law in psychophysics reveals a functional relationship in which the concept membership value of the subjective response is proportional to the logarithm value of the acceleration:

$$v\left(u\right)=a\ln\left(u\right)+b.$$

It can be seen from the aforementioned formula that when we know the perceptible limit and tolerance limit of the human body’s response in a certain vibration environment, we can use the aforementioned formula to obtain the value of the membership degree of the crowd’s annoyance response.

According to the theory of psychophysical signal detection, there are two main steps in the human response to vibration: perceiving vibration and judging vibration. Among them, people’s perception of vibration is uncertain, and at the same time, people’s subjective judgment of vibration is ambiguous. For the case of continuous distribution, the difference in sensitivity can be described by the lognormal distribution function:

$$f\left(a_{w}|u\right)=\frac{1}{\sqrt{2\pi }u\sigma }\exp\left\{\frac{-{\left[\ln\left(u\right)-\mu_{\ln\left(a_{w}\right)}\right]}^{2}}{2\sigma^{2}}\right\},$$

where $\sigma^{2}=\ln\left(1+\delta^{2}\right)$ , $\mu_{\ln\left(a_{w}\right)}=\ln\left(a_{w}\right)-\sigma^{2}/2$ , $a_{w}$ , and σ are the expected value and variation function of u , respectively, and δ is the variation function of random normal distribution. According to the research of many scholars, the value of δ is generally 0.2-0.5, and the value of 0.3 is used in this paper to study the human annoyance rate.

Taking into account the difference between the human body in a wheelchair and other vibration environments, the calculation formula of the annoyance rate value of the vibration acceleration $a_{w}$ when the whole wheelchair is taken is given as follows:

$$A\left(a_{w}\right)=\int_{u_{\min}}^{\infty }\frac{1}{\sqrt{2\pi }u\sigma }\exp\left\{\frac{-{\left[\ln\left(u/a_{w}\right)+0.5\sigma^{2}\right]}^{2}}{2\sigma^{2}}\right\}v\left(u\right)du,$$

where $v\left(u\right)$ is a membership function and $u_{\min}$ is the lower limit of acceleration corresponding to the perceptible limit of vibration.

The annoyance rate under a random vibration intensity $a_{w}$ can be calculated by the aforementioned formula. According to the definition of fuzzy membership function, the acceleration r1 corresponding to the perceptible limit and the acceleration r2 corresponding to the tolerance limit are known; the coefficients a and b of the membership function can be calculated, and then the annoyance rate value can be obtained by integrating according to the annoyance rate calculation formula, and an annoyance rate curve can be drawn.

$$\left\{\begin{array}{l} a\ln\left(r1\right)+b=0\\ a\ln\left(r2\right)+b=1 \end{array}\right..$$

According to the evaluation table of ISO-2631-1, the human body’s perceptible limit (the lower limit of subjective response) during system driving is $0.315m/s^{2}$ , the tolerance limit (the upper limit of subjective response) is $2.5m/s^{2}$ , $r1=0.315m/s^{2}$ , and $r2=2.5m/s^{2}$ , and thus we get a=0.48 , b=0.56 . According to the aforementioned annoyance rate formula, the human annoyance rate curve in the driving environment can be obtained, as shown in Figure 2B.

## 4 Wheelchair–biomechanical model of the human body system

### 4.1 Establishment of the human biomechanical model

According to GB-10000-88 “Human Dimensions of Chinese Adults” and related theories of bionics, the height of Chinese adults is selected as 1,800 mm, the weight as 65 kg, and the percentage as 50 (that is, in certain sample data, it is higher or lower than the height and weight data) Accounting for 50%, these data are applied to LifeMOD to build a human mechanics model and define the parameters of each joint. The mechanical parameters of human joints adopt the joint mechanical parameters obtained through the actual testing of the Hybrid III crash dummy, mainly including non-linear stiffness, damping and friction values, and limiting joint stiffness with hysteresis. After the human body model is established, the posture adjustment function is used to adjust the angle of the human skeleton so that the sitting posture of the human body completely conforms to the posture of riding a wheelchair in reality.

When using anthropometric parameters or demographic parameters to create a virtual human body, ADAMS uses the GeBOD program to calculate the necessary parameters. The GeBOD program outputs data describing the human body model of the 15-segment rigid body. The position of the axilloid, the joint that connects the links. The anthropometric data calculated by GeBOD is based on 32 default morphometric parameters. If no updated values for these parameters are provided, GeBOD will output the default values for the parameters. Table 2 shows the output of default data by the human body model in this paper:

**TABLE 2:** Human body model output of default data.

Project name	Human data (mm)
Ankle circumference	211.0435825
Ankle height, outside	140.4683459
Armpit height	1323.854381
Biceps circumference	280.3974815
Buttock depth	209.271062
Calf circumference	341.0846869
Chest breadth	301.1401516
Chest depth	218.5973197
Elbow circumference	299.4991444
Foot breadth	95.97301467
Foot length	270.7387811
Forearm circumference	262.5570257
Forearm/head length	499.5140956
Head breadth	153.6571
Head length	197.4350064
Head to chin height	228.946798
Hip breadth, standing	331.105377
Knee circumference	367.3743512
Neck circumference	360.1179666
Knee height, seated	562.4855635
Seated height	938.988042
Shoulder breadth	479.1378574
Shoulder height	1468.070504
Shoulder-to-elbow length	365.0986821
Standing height	1,800
Thigh circumference	519.2038789
Upper leg circumference	361.9016057
Waist breadth	275.0127284
Waist depth	191.4139039
Waist height	1089.26063
Ankle circumference	168.8972904

The GeBOD program is used to calculate the body posture parameters and establish the human body model. The regression equations of the shape parameters of the human body model, body segment mass, body segment centroid, and overall centroid position are shown in the following formula:

$$Y=B_{0}+B_{1}X_{1}+B_{2}X_{2}+B_{3}X_{3},$$

where Y is the mass or centroid; $B_{0}$ is the constant term of the regression equation; $B_{1}$ is the weight regression coefficient; $X_{1}$ is the weight; $B_{2}$ is the regression coefficient for height; $X_{2}$ is the height; $B_{3}$ is the regression coefficient for age; and $X_{3}$ is the age.

Based on the aforementioned formula, the body segment mass of the human body model and the moment of inertia of the center of mass of the single segment can be obtained. The mass and moment of inertia of each body segment of the human body model established in this paper are shown in Table 3:

**TABLE 3:** Mass and moment of inertia of each body segment of the human body model.

Parts	Quality (Kg)	Ixx (Kg*mm ^ 2)	Iyy (Kg*mm ^ 2)	Izz (Kg*mm ^ 2)
Head	4.929	2.98E+04	2.61E+04	1.54E+04
Left_Foot	2.076	9.68E+03	8.58E+03	3.00E+03
Left_hand	0.407	4.62E+02	4.62E+02	2.30E+02
Left_Lower_Arm	1.398	9.17E+03	9.17E+03	9.74E+02
Left_Lower_Leg	3.465	4.87E+04	4.87E+04	4.08E+03
Left_Scapula	1.773	5.55E+03	5.55E+03	2.51E+03
Left_Upper_Arm	1.662	1.19E+04	1.19E+04	1.32E+03
Left_Upper_Leg	5.807	8.45E+04	8.45E+04	1.14E+04
Lower_Torso	7.496	5.75E+04	5.47E+04	2.99E+04
Neck	1.208	2.39E+03	2.39E+03	1.58E+03
Right_Foot	2.076	9.68E+03	8.58E+03	3.00E+03
Righr_Hand	0.407	4.62E+02	4.62E+02	2.30E+02
Right_Lower_Arm	1.398	9.17E+03	9.17E+03	9.74E+02
Right_Lower_Leg	3.465	4.87E+04	4.87E+04	4.08E+03
Right_Scapula	1.773	5.55E+03	5.55E+03	2.51E+03
Right_Upper_Arm	1.662	1.19E+04	1.19E+04	1.32E+03
Right_Upper_Leg	5.807	8.45E+04	8.45E+04	1.14E+04
Upper_Torso	12.746	1.32E+05	1.05E+05	8.82E+04

### 4.2 Wheelchair–human body system model

**FIGURE 3:**
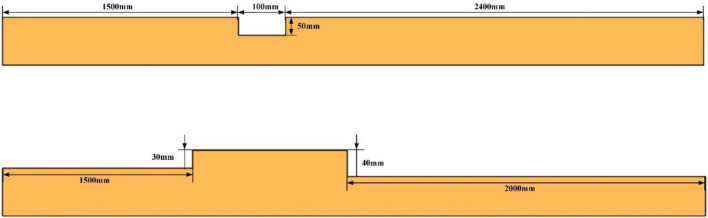
Sectional view of ditch pavement and step pavement.

In this paper, the road surface file adopts the “rdf” file format and imports it into the simulation environment through the “tire” module, and its essence is still the structure of the surface file that is still an ASCII text file in the “TeimOrbit” format. The XY axis is the plane of the road surface, and the *Z*-axis is the height of the road surface. When making a rigid pavement, first use 3D software to draw the required plane, divide the pavement into a triangular mesh, and then export it to a node format file, which contains information on each node and grid unit in the pavement model. The information to the road surface file template is copied, and the road surface adhesion coefficient is set to get the rigid road surface we need. In this paper, we have established two kinds of rigid pavements: ditch road and step road. The schematic diagrams of the two roads are shown in Figure 3.

Based on the wheelchair, human body, and rigid road model established previously, the model integration function in the LifeMod plug-in is used to establish a comprehensive model of the “wheelchair–human body-road,” as shown in Figure 3. We set the speed of the wheelchair to drive on the simulated road. Therefore, the existing wheelchair platform used in this paper is the front-wheel drive, and the rotation speed of the front wheel is set to 1.57 rad/s (0.28 m/s).

## 5 Simulation and experimental verification

### 5.1 Test condition setting

In order to verify the stability and comfort of the wheelchair–human body system on the aforementioned road surface, this paper modifies the existing intelligent wheelchair prototype platform and builds a test road for the wheelchair–human body system driving test. In order to be able to control the wheelchair platform to drive on the test road while the test personnel are in a completely relaxed state, a control system for the wheelchair platform is built in this paper. Among them, the lower control system uses the STM32 single-chip microcomputer to control the wheelchair front wheel motor to make the wheelchair platform move, and the upper computer system uses the Huayan industrial computer-integrated ROS development system for control input and data processing.

During the driving process of the wheelchair, this paper uses the inertial measurement unit to collect real-time data on the position of the wheelchair frame and the acceleration and angle of the human head and chest during the test and compares it with the simulation analysis to verify the accuracy of the simulation method in this paper. In order to ensure the consistency between the test conditions and the simulation conditions, cement is used to make the test ditch and step road surface. The length of the front section of the ditch ridge is 2,500 mm, the width of the ditch ridge is 100 mm, the depth is 50 mm, and the length of the rear section is 1,400 mm. The length of the front end is 1,400 mm, the height of the front end of the step obstacle is 40 mm, the length of the step is 700 mm, the height of the back section of the step is 30 mm, and the length of the back section is 2,000 mm. In the road driving test, the testers first drove the wheelchair through the ditch and ridge obstacles, then through the step obstacles, and stopped the test after passing the step obstacles smoothly. During the test, the wheelchair speed was maintained at a constant speed of 0.28 m/s, which was consistent with the wheelchair speed in the simulation analysis.

### 5.2 Comparison of results of the ditch pavement

**FIGURE 4:**
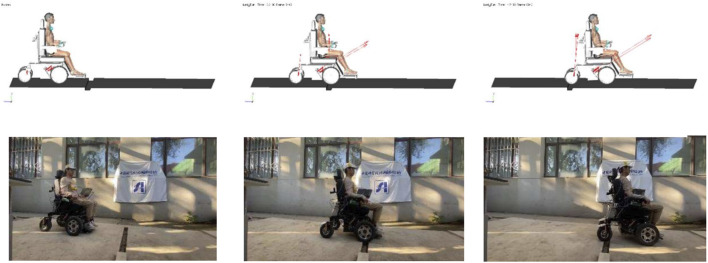
Schematic diagram of the ditch pavement test.

According to the basic working conditions set by the simulation, the wheelchair prototype is used as the test equipment, and the test personnel wear the sensor to drive the wheelchair through the ditch road and analyze the stability and comfort of the wheelchair–human system during the process. First, the wheelchair is parked at the end of the front section of the ditch and ridge road, and the wheelchair speed is set for the test. After passing through the ditch road and stopping smoothly, the acceleration and angle data are recorded by the sensors worn on the tester’s head and chest. Figure 4 shows the comparison between the simulation and the test under the ditch road.

**FIGURE 5:**
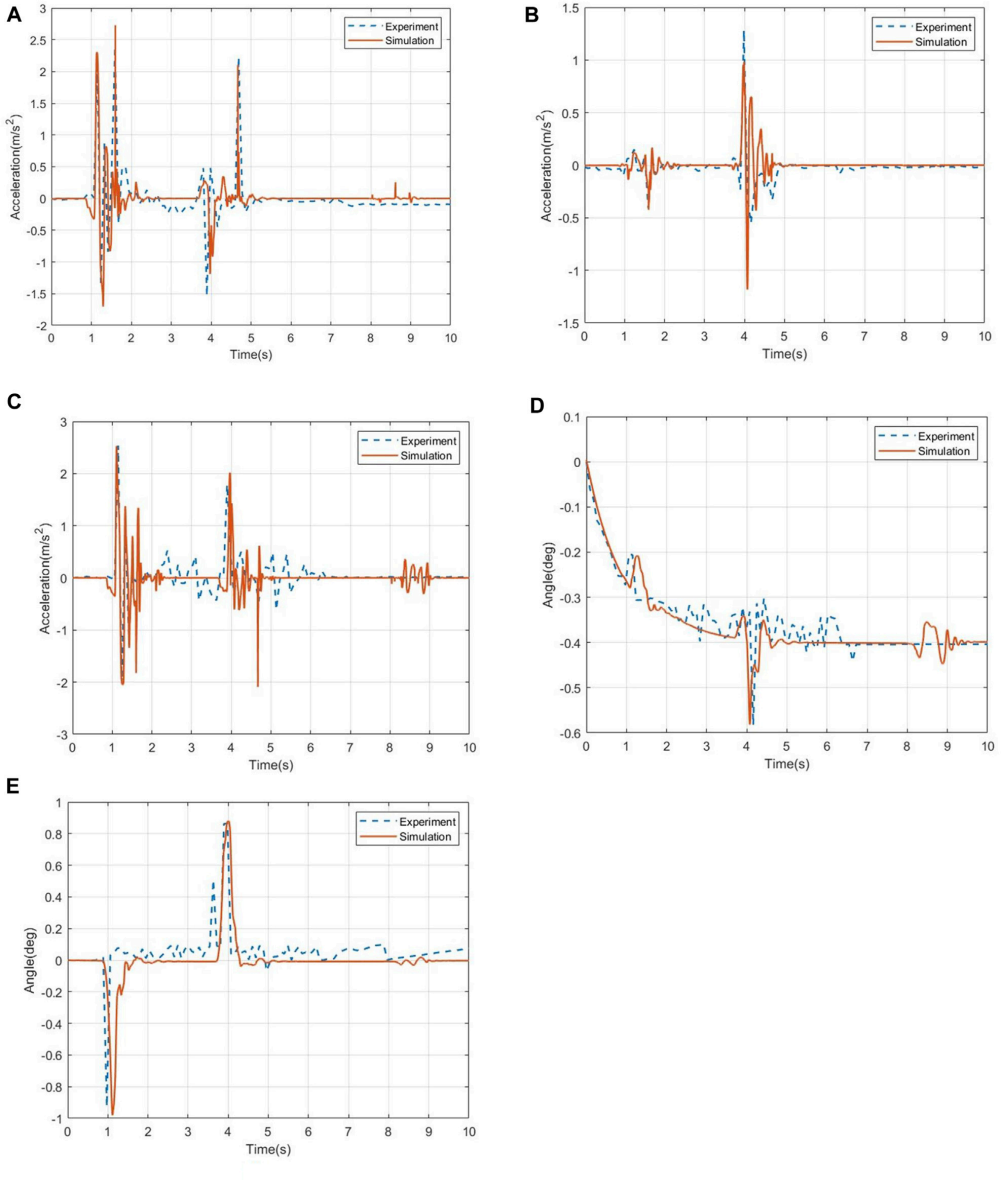
Comparison of the acceleration data and angle change of the wheelchair frame on the ditch road. **(A)** Acceleration in the pitch direction. **(B)** Acceleration in the roll direction. **(C)** Acceleration in the vertical direction. **(D)** Roll angle change of the wheelchair frame position. **(E)** Pitch angle change of the wheelchair frame position.

The comparison of the acceleration data of the wheelchair frame under the ditch road is shown in Figures 5A–C, where A is the acceleration in the roll direction, B is the acceleration in the pitch direction, and C is the acceleration in the vertical direction. In Figures 5A–C, it can be seen from the acceleration data that the results show that the data error of the wheelchair pitch acceleration is 2.25%, the data error of the roll acceleration is 1.73%, and the data error of the vertical acceleration is 6.17%. The test and simulation data show better findings.

Figures 5D, E show the data comparison of the angle change of the wheelchair under the ditch road. The results show that the data error of the roll angle of the wheelchair is 5.46%, and the data error of the pitch angle is 4.73%, both of which are within the acceptable range, which verifies the accuracy of the simulation method used in this paper.

**FIGURE 6:**
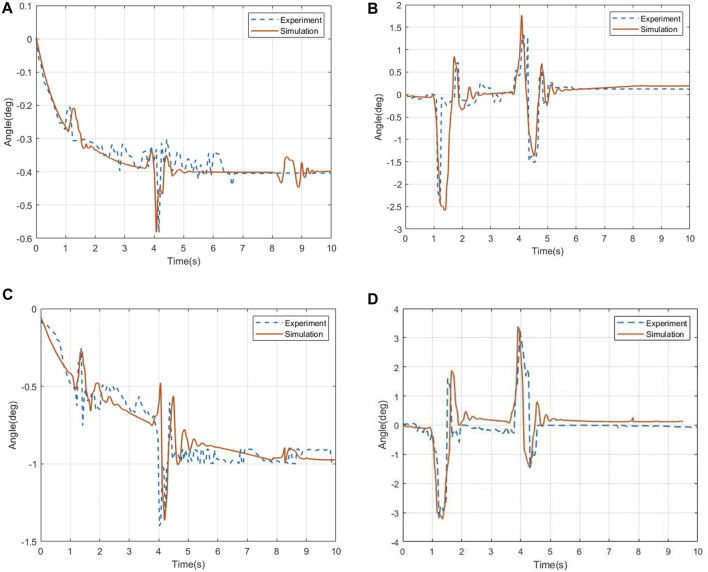
Changes in the angle data of the human chest and head. **(A)** Roll angle of the human chest. **(B)** Pitch angle of the human chest. **(C)** Roll angle of thehuman head. **(D)** Pitch angle of the human head.

The comparison of the roll angle and pitch angle data of the human chest and head under the ditch road is shown in Figure 6, where Figures 6A, B show the comparison of the angle data of the measurement point of the human chest under the road surface of the ditch, and Figures 6C, D show the measurement point of the human head in the ditch. Angle data comparison under the rough road is as follows: in terms of data errors, the errors of the roll angle and pitch angle of the human chest are 1.65% and 1.93%, respectively, and the errors of the roll angle and pitch angle of the human head are 1.85% and 1.62%, respectively.

**FIGURE 7:**
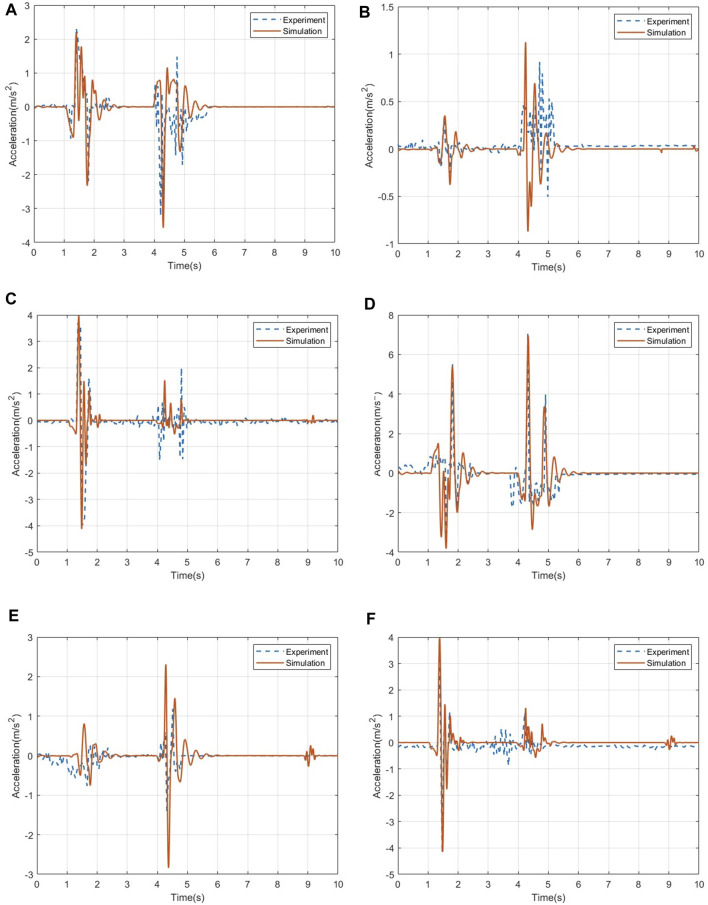
Comparison of acceleration data of the human chest and head. **(A)** Acceleration in the pitch direction of the human chest. **(B)** Acceleration in the roll direction of the human chest. **(C)** Acceleration in the vertical direction of the human chest. **(D)** Acceleration in the pitch direction of the human head. **(E)** Acceleration in the roll direction of the human head. **(F)** Acceleration in the vertical direction of the human head.

For the acceleration data, Figure 7 shows the three-axis acceleration data of the human chest and head under the ditch road. In the acceleration response of the human chest, the data errors in the three directions are 5.59%, 8.71%, and 4.65%. Through the comparison of the acceleration data of the head, it can be seen that the pitch acceleration of the head in the test is significantly higher than that of the chest of the human body, indicating that the head swings back and forth more violently during the test. After that, the acceleration basically returned to a stable level. Calculation of the error shows that the pitch acceleration data error of the human head is 6.25%, the roll acceleration data error is 6.82%, and the vertical acceleration data error is 3.33%. The simulation data of the head and chest fit well, and the change trend of the data is consistent with the simulation analysis in this paper.

According to the comfort evaluation index in this paper, we calculate the root-mean-square value of the acceleration of the human chest and head under the ditch road surface and weigh it to evaluate the comfort of the human body. Table 4 is the evaluation form of the human chest and head. According to the calculation results, it can be seen that the total vibration of the human chest under the ditch road surface is 0.1891, and the annoyance rate is 0.18%. The total vibration of the human head under the road surface is 0.2932, and the annoyance rate is 3.48%. That is, the chest and head of the human body are kept in a good comfortable state during the process.

**TABLE 4:** Human comfort evaluation form on the ditch pavement.

	Human chest	Human head
Total vibration (RMS)	0.1891	0.2932
Annoyance rate (%)	0.18%	3.48%

### 5.3 Comparison of the result of the step pavement

**FIGURE 8:**
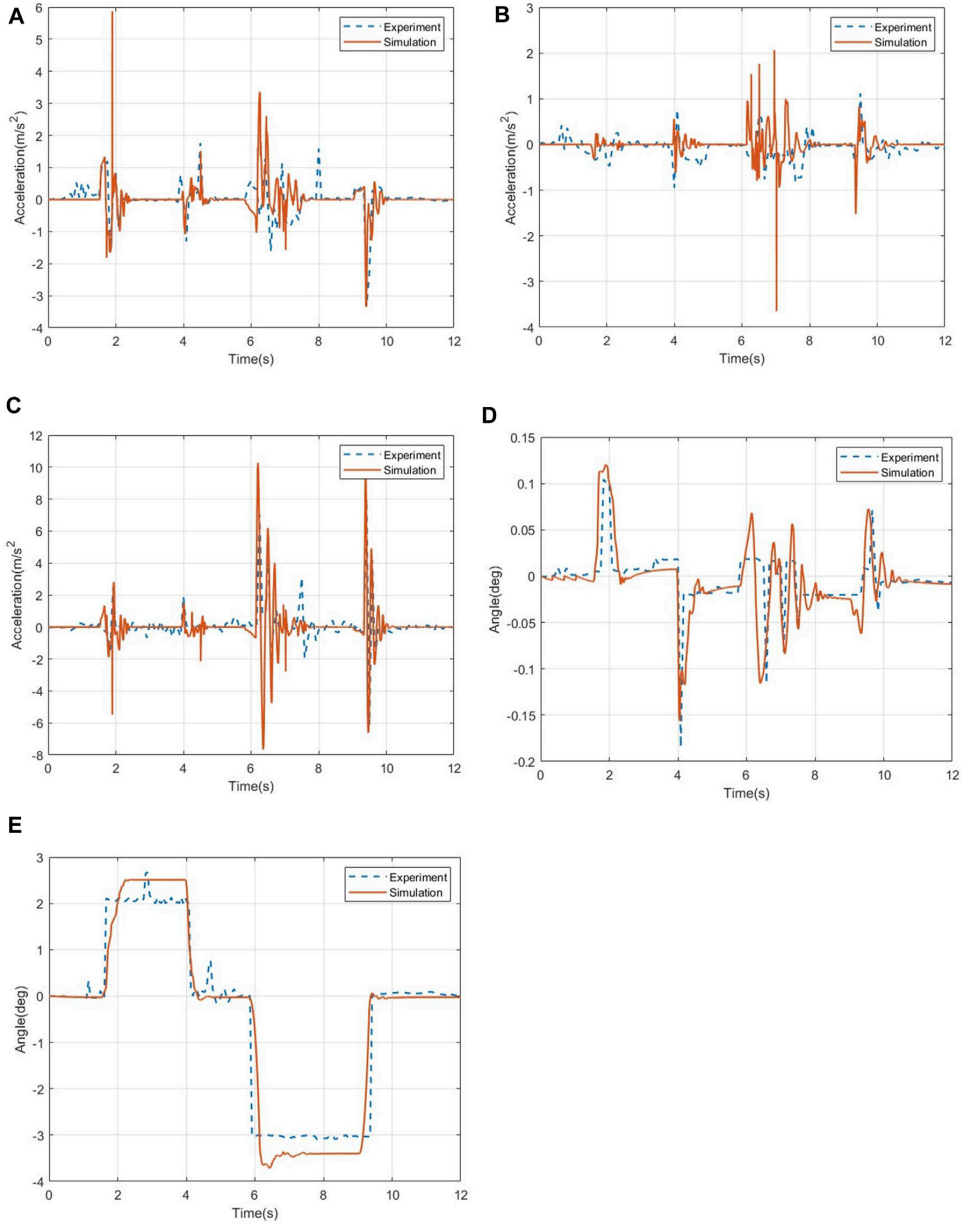
Comparison of the acceleration data and angle change of the wheelchair frame on the step road. **(A)** Acceleration in the pitch direction. **(B)** Acceleration in the roll direction. **(C)** Acceleration in the vertical direction. **(D)** Roll angle change of the wheelchair frame position. **(E)** Pitch angle change of the wheelchair frame position.

According to the setting of the simulation working conditions, the driving speed of the wheelchair is set to 1.57 rad/s (0.28 m/s). After the test personnel have adjusted their sitting posture, the test starts. Through the wheelchair frame position recorded by the sensor, the acceleration and angle data of the human chest and head are compared with the test data of the wheelchair–human body system on the road surface and compared with the simulation results. Figure 8 shows the comparison between simulation and test results under the stepped pavement.

The comparison of the acceleration data of the wheelchair frame on the step road is shown in Figures 8A–C. From the comparison of the acceleration data, it can be seen that the acceleration change trend of the wheelchair frame obtained in the step test is very close to the peak point. The data results show the acceleration in the pitch direction of the wheelchair. The data error is 3.24%, the acceleration data error in the roll direction is 9.62%, and the acceleration data error in the vertical direction is 3.51%.

Figures 8D, E show the data comparison of the angle change of the wheelchair under the step road. The results show that the data error of the roll angle of the wheelchair is 8.32%, and the data error of the pitch angle is 2.25%, both of which are within the acceptable range, which verifies the accuracy of the simulation method used in this paper.

**FIGURE 9:**
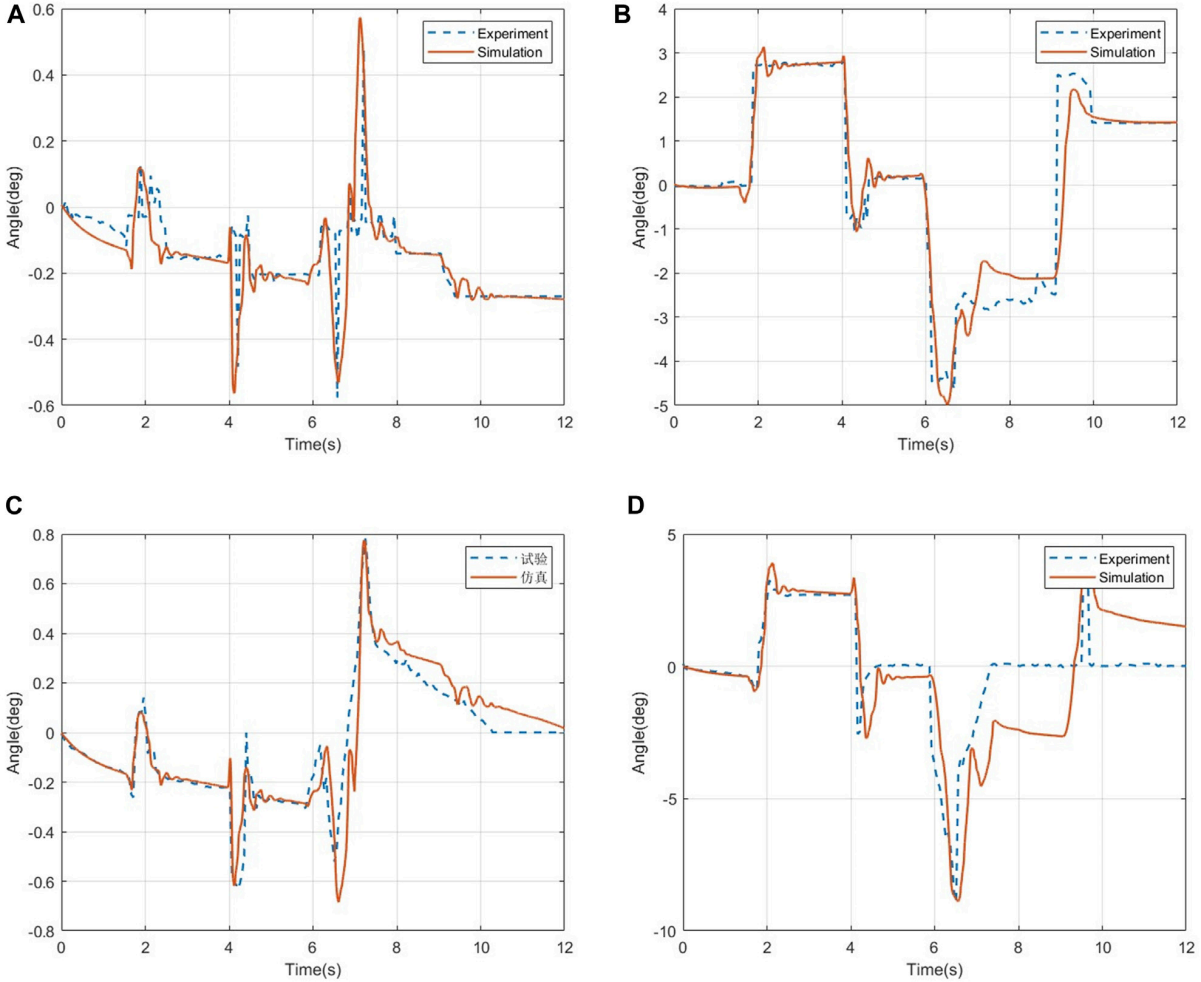
Changes in the angle data of the human chest and head. **(A)** Roll angle of the human chest. **(B)** Pitch angle of the human chest. **(C)** Roll angle of the human head. **(D)** Pitch angle of the human head.

The comparison of the roll angle and pitch angle data of the human chest and head under the step road is shown in Figure 9, in which Figures 9A, B show the comparison of the angle data of the human chest measurement point under the step road, and Figures 9C, D show the comparison of the angle data of the head measurement points under the step road. In terms of data errors, the roll angle and pitch angle errors of the human chest are 5.58% and 3.62%, respectively, and the roll angle and pitch angle errors of the human head are 2.87% and 4.35%, respectively.

**FIGURE 10:**
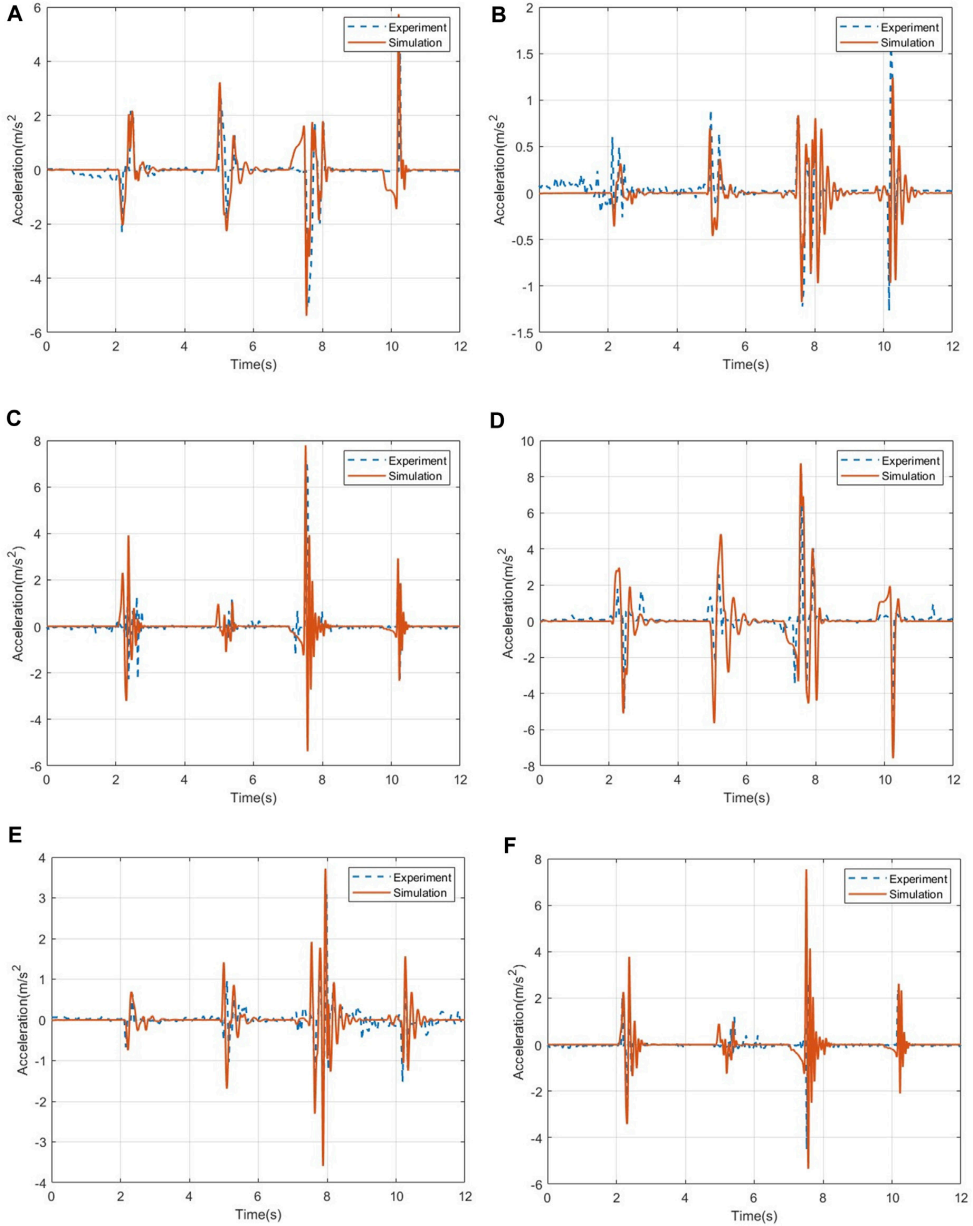
Acceleration data of the human chest and head under the step pavement. **(A)** Acceleration in the pitch direction of the human chest. **(B)** Acceleration in the roll direction of the human chest. **(C)** Acceleration in the vertical direction of the human chest. **(D)** Acceleration in the pitch direction of the human head. **(E)** Acceleration in the roll direction of the human head. **(F)** Acceleration in the vertical direction of the human head.

In the acceleration data, Figure 10 shows the three-axis acceleration data of the human chest and head under the step road. In the acceleration response of the human chest, the data errors in the three directions are 4.13%, 4.59%, and 5.27%. Through the comparison of the acceleration data of the head, it can be seen that the pitch acceleration of the head in the test is significantly higher than that of the chest of the human body, indicating that the head swings back and forth more violently during the test. After that, the acceleration basically returned to a stable level. Calculation of the error shows that the pitch acceleration data error of the human head is 3.28%, the roll acceleration data error is 4.16%, and the vertical acceleration data error is 2.65%, the simulation data of the head and chest fit well, and the change trend of the data is consistent with the simulation analysis in this paper.

According to the calculation of the comfort evaluation index, the root-mean-square values of the acceleration of the human chest and head under the step road are 0.2531 and 0.2975, respectively, and the corresponding annoyance rates are 1.54% and 3.74%, respectively, as shown in Table 5. All are less than the critical value corresponding to the comfort evaluation system in this paper. It can be judged that the chest and head of the human body have maintained a good comfortable state during the process of passing the obstacle, and the human body has good comfort when passing the obstacle road.

**TABLE 5:** Human comfort evaluation form on the ditch pavement.

	Human chest	Human head
Total vibration (RMS)	0.2531	0.2975
Annoyance rate (%)	1.54%	3.74%

## 6 Conclusion

(1) Based on the traditional vehicle stability theory, this paper conducts a mechanical analysis of the wheelchair–human body system in rollover and pitch states. Then, the expressions of the limit roll angle and pitch angle of the system were obtained during dynamic driving.
(2) The ISO-2631-1 standard and the subjective psychological annoyance rate were used to establish the objective and subjective comfort evaluation systems of the human body. A theoretical basis for system stability and comfort evaluation was provided in subsequent simulations and experiments.
(3) Based on the ADAMS/LifeMOD biomechanics plug-in and the existing wheelchair prototype, a biomechanical model of the wheelchair–human body system and the wheelchair–human body-rigid road model of the ditch road surface and the step road surface were established, and the model integration function in the lifeMOD plug-in was used to establish the “wheelchair–human body road” model of the ditch road surface and the step road surface, respectively.
(4) Through the transformation of the existing wheelchair prototype platform and the construction of the test road, the wheelchair–human body system was tested and verified under the same working conditions. By obtaining and comparing the experimental and simulation data, the results showed that the position of the wheelchair frame, the angular velocity and acceleration of the human chest and head, and the error of the test data and the simulation analysis are all within 10%, which fully demonstrates the accuracy of the multi-body dynamics method used in this paper. The result shows that the peak acceleration of the head in three directions is higher than that of the chest data. This is because the head has less support throughout the ride, causing the head to swing more dramatically than the chest during the ride.
(5) The stability and comfort of the system under the ditch and step roads were evaluated. The stability and comfort of the system under the ditch and step roads are evaluated by the data obtained from the test. The results show that the wheelchair–human system remains in a good stable and comfortable state during driving on the two road surfaces.

## Data Availability

The original contributions presented in the study are included in the article/Supplementary Material; further inquiries can be directed to the corresponding author.
